# American Surgical Association

**Published:** 1896-06

**Authors:** 


### AMERICAN SURGICAL ASSOCIATION.

The recent meeting of this body at Detroit, Mich., on the 26th, 27th and 28th of May, was attended by Dr. J. McFadden Gaston, of this city. The programme was of great interest, and Dr. Gaston was assigned to open the discussion upon the surgical treatment of Tuberculosis, while he filled a more important role in presenting a paper upon "An Improved Method of Exploring the Thoracic cavity." A cadaver was used for demonstrating the process of opening a trap-door into the chest, by cutting through the ribs from the third to the seventh in the axillary line, and making transverse incisions forwards from the upper and lower extremities of this line to the costal cartilages. By elevating the free margin of this flap upon the hinge formed by the cartilaginous attachment of the ribs, the entire contents of the left side of the chest came into view.

The comments upon this mode of access to the thoracic cavity from various prominent fellows of the Asociation indicated their appreciation of this modification of Delorme's plan for entering the thorax; and Dr. Nicholas Senn said to Dr. Gaston that he expected to avail himself of it at an early day.

The place of meeting of The American Surgical Association for the coming year, will be Washington, D. C., in the month of May, when the statue of Dr. S. D. Gross will be unveiled, with an address upon his life and character by Dr. W. W. Keen, of Philadelphia.

The officers elected for the ensuing year, are, for President, Dr. J. Collins Warren, Cambridge, Mass.; First Vice-President, Dr. Thos. A. McGraw, Detroit, Mich.; Second Vice-President, Dr. John Ashhurst, Jr., Philadelphia, Pa.; Secretary, Dr. Maurice

H. Richardson, Boston, Mass.; Treasurer, Dr. N. P. Dandridge, Cincinnati, Ohio.

During the meeting a boat excursion on the lake, with a sumputous entertainment, was extended to the members of the Association and their families, by Dr. McGraw, as chairman of the Committee of Arrangements.

